# Transcriptome Analysis in Rat Kidneys: Importance of Genes Involved in Programmed Hypertension

**DOI:** 10.3390/ijms16034744

**Published:** 2015-03-02

**Authors:** You-Lin Tain, Li-Tung Huang, Julie Y. H. Chan, Chien-Te Lee

**Affiliations:** 1 Department of Pediatrics, Kaohsiung Chang Gung Memorial Hospital and Chang Gung University College of Medicine, Kaohsiung 833, Taiwan; E-Mail: litung.huang@gmail.com; 2 Center for Translational Research in Biomedical Sciences, Kaohsiung Chang Gung Memorial Hospital and Chang Gung University College of Medicine, Kaohsiung 833, Taiwan; E-Mail: jchan@adm.cgmh.org.tw; 3 Department of Traditional Chinese Medicine, Chang Gung University, Linkow 244, Taiwan; 4 Division of Nephrology, Departments of Medicine, Kaohsiung Chang Gung Memorial Hospital and Chang Gung University College of Medicine, Kaohsiung 833, Taiwan; E-Mail: ctlee33@adm.cgmh.org.tw

**Keywords:** arachidonic acid, developmental programming, fructose, glucocorticoid, hypertension, next generation sequencing, nitric oxide

## Abstract

Suboptimal conditions in pregnancy can elicit long-term effects on the health of offspring. The most common outcome is programmed hypertension. We examined whether there are common genes and pathways in the kidney are responsible for generating programmed hypertension among three different models using next generation RNA sequencing (RNA-Seq) technology. Pregnant Sprague-Dawley rats received dexamethasone (DEX, 0.1 mg/kg) from gestational day 16 to 22, 60% high-fructose (HF) diet, or N^G^-nitro-l-arginine-methyester (l-NAME, 60 mg/kg/day) to conduct DEX, HF, or l-NAME model respectively. All three models elicited programmed hypertension in adult male offspring. We observed five shared genes (*Bcl6*, *Dmrtc1c*, *Egr1*, *Inmt*, and *Olr1668*) among three different models. The identified differential genes (DEGs) that are related to regulation of blood pressure included *Aqp2*, *Ptgs1*, *Eph2x*, *Hba-a2*, *Apln*, *Guca2b*, *Hmox1*, and *Npy*. RNA-Seq identified genes in arachidonic acid metabolism are potentially gatekeeper genes contributing to programmed hypertension. In addition, HF and DEX increased expression and activity of soluble epoxide hydrolase (*Ephx2* gene encoding protein). Conclusively, the DEGs in arachidonic acid metabolism are potentially gatekeeper genes in programmed hypertension. The roles of DEGs identified by the RNA-Seq in this study deserve further clarification, to develop the potential interventions in the prevention of programmed hypertension.

## 1. Introduction

Embryonic and fetal periods are vulnerable to environmental factors. Hypertension is a highly prevalent disease and it may come from early life [1]. The kidney has been identified as a key player in the development of hypertension, mainly by reduced nephron number, impaired natriuresis, activation of vasoconstriction, and oxidative stress [2,3]. Adverse intrauterine environment during critical periods of kidney development can elicit epigenetic alterations leading to renal programming and developing hypertension in adult life—namely developmental programming [4].

We recently observed that programmed hypertension developed in the male offspring of rats exposed a variety of insults including maternal caloric restriction [5,6], diabetes [7], high fructose (HF) diet [8], nitric oxide (NO) inhibition [9], and dexamethasone (DEX) treatment [10,11]. A number of mechanisms, including glucocorticoid effects, oxidative stress, epigenetic regulation, alterations of renin-angiotensin system (RAS) and sodium transporters, and reduction in nephron numbers, have been demonstrated to interpret programmed hypertension [4,12]. However, each of the proposed mechanisms examined in different developmental programming models was unable entirely determine the common underlying mechanisms that drive programmed hypertension process. Currently, no genome-wide study has been conducted to capture commonality in transcriptional regulatory gene networks shared by various prenatal insults in the kidney with similar hypertension phenotype. Therefore, we employed the whole-genome RNA next-generation sequencing (RNA-Seq) to quantify the abundance of RNA transcripts in the offspring kidneys from maternal exposure to DEX, HF, or N^G^-nitro-l-arginine-methyester (l-NAME, an inhibitor of NO synthase). The aim of this research was to determine whether there is a gatekeeper gene or pathway in the kidney, which is responsible for generating programmed hypertension among these three different models. A clearer understanding of the similarities and differences of regulated genes between diverse programming models is essential to improve therapeutic strategy and prevent the development of hypertension in the offspring exposed to suboptimal conditions during pregnancy.

## 2. Results

### 2.1. Hypertension Is a Common Phenotype in Response to DEX, HF, and l-NAME

**Figure 1:**
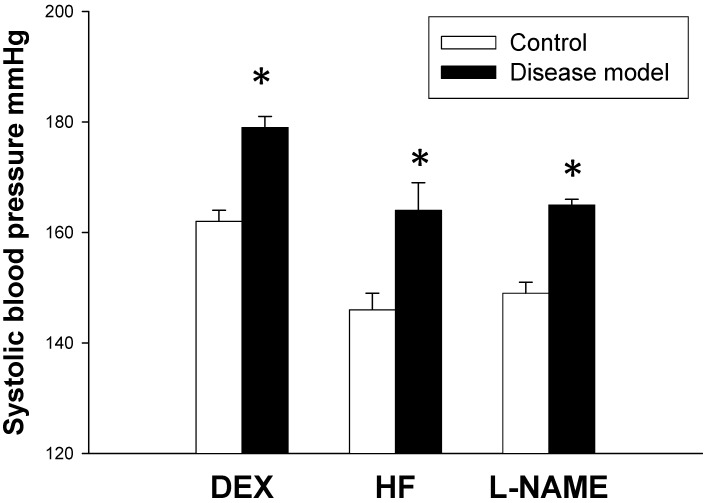
Effects of dexamethasone (DEX), high-fructose intake (HF), and N^G^-nitro-l-arginine-methyester (l-NAME) on systolic blood pressure in male adult offspring. *n* = 6 per group. * *p* < 0.05 *vs.* control.

As shown in Figure 1, the systolic blood pressure (BP) of DEX group was significantly greater than that of control at 16 weeks of age. Similarly, HF and l-NAME group had greater systolic BP than control at 12 weeks of age. These data have been published previously [8,9,10].

### 2.2. The Effects of DEX, HF, and l-NAME on Renal Transcriptome

**Figure 2:**
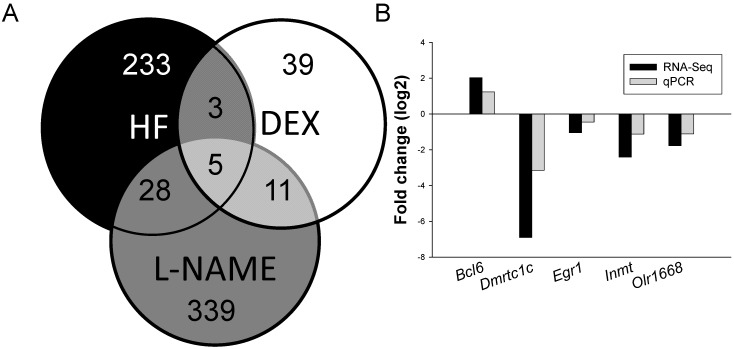
(**A**) Venn diagram depicting unique and shared (overlapping circles) sets of DEGs between high-fructose intake (HF, black circle), dexamethasone treatment (DEX, white circle), and l-NAME treatment (grey circle); (**B**) Confirmatory analysis of kidney gene expression by qPCR in the l-NAME model. Individual gene expression was determined by qPCR and expressed as fold change in log base 2 *vs.* control and graphed alongside fold change derived from RNA-Seq analysis.

We next analyzed differential gene expression induced by three different insults in the offspring kidney. The mappability of genes in each group compared to the rat reference genome was 79.93% for DEX group, 87.15% for HF group, and 83.31% for l-NAME group, respectively. Among the differential expressed genes (DEGs), a total of 58 genes (Table S1, 35 up- and 23 down-regulated genes by DEX *vs.* control) met the selection criteria of (1) genes that changed by reads per kilobase of transcript per million mapped reads (RPKM) >0.3 and (2) minimum of twofold difference in normalized read counts between group. *p* value was estimated for each gene and corrected for multiple testing by the Benjamini-Hochberg correction. The log2 fold change (FC) was used to partition the genes into up- and down-regulated groups. Next, 269 DEGs (Table S2, 197 up- and 72 down-regulated genes) was noted in HF *vs.* control [8]. We observed 383 DEGs (Table S3, 198 up- and 185 down-regulated genes by l-NAME *vs.* control) in response to NO inhibition [13]. Genes shared by different insults are represented graphically by the Venn diagram shown in Figure 2A.

Among them, a total of five shared genes (*Bcl6*, *Dmrtc1c*, *Egr1*, *Inmt*, and *Olr1668*) were identified among DEX *vs.* control, HF *vs.* control, and l-NAME *vs.* control group (Table 1). These five genes were quantified by qPCR to validate RNA-Seq data. The results thus obtained were in agreement with deep-sequencing data (Figure 2B). Next, we observed that 8 DEGs are shared between HF and DEX, 33 DEGs between HF and l-NAME, and 16 DEGs between DEX and l-NAME (Table 1).

**Table 1:** Changes of shared differential expressed genes (DEGs) between DEX, HF, and l-NAME group in the adult offspring kidney.

Gene ID	Gene Symbol		Fold Changes	
**3 groups**		**HF**	**DEX**	**l-NAME**
ENSRNOG00000001843	*Bcl6*	5.401	0.217	3.277
ENSRNOG00000037923	*Dmrtc1c*	12.62	38.46	0.018
ENSRNOG00000019422	*Egr1*	2.885	3.947	0.480
ENSRNOG00000011250	*Inmt*	0.283	0.275	0.188
ENSRNOG00000029622	*Olr1668*	4.562	4.106	0.060
**2 groups: HF and DEX**		**HF**	**DEX**	
ENSRNOG00000012531	*Ephb2*	2.254	5.554	
ENSRNOG00000017286	*Ephx2*	24.34	16.76	
ENSRNOG00000029651	*Rdh2*	0.228	0.195	
**2 groups: HF and l-NAME**		**HF**		**l-NAME**
ENSRNOG00000005542	*Apob*	0.254		2.167
ENSRNOG00000007830	*Apold1*	2.658		0.369
ENSRNOG00000003745	*Atf3*	4.408		0.294
ENSRNOG00000043249	*B0BNK5_RAT*	2.148		0.345
ENSRNOG00000021027	*Dbp*	4.491		4.966
ENSRNOG00000000577	*Ddit4*	2.319		2.003
ENSRNOG00000016301	*Dmrt2*	2.361		2.061
ENSRNOG00000003977	*Dusp1*	2.379		0.380
**2 groups: HF and l-NAME**		**HF**		**l-NAME**
ENSRNOG00000029919	*F1M7Y9_RAT*	4.44 × 10^−4^		8.03 × 10^−6^
ENSRNOG00000011631	*Fst*	4.452		0.403
ENSRNOG00000013090	*Gadd45g*	2.732		0.156
ENSRNOG00000026053	*GREM1_RAT*	2.798		2.093
ENSRNOG00000003616	*Grem2*	3.557		9.922
ENSRNOG00000015724	*Gucy2g*	46.59		7.268
ENSRNOG00000029886	*Hba-a2*	2.009		0.131
ENSRNOG00000033465	*Hbb*	2.608		0.049
ENSRNOG00000019120	*Hmgcs2*	0.318		3.421
ENSRNOG00000010999	*K1731_RAT*	0.428		2.833
ENSRNOG00000032857	*Klks3*	0.284		0.233
ENSRNOG00000012871	*LOC100360318*	26291		153519
ENSRNOG00000030492	*LOC100364577*	0.199		0.135
ENSRNOG00000013872	*P2ry14*	3.641		2.316
ENSRNOG00000018187	*Racgap1*	3.481		2.055
ENSRNOG00000005447	*RGD1311564*	0.494		0.443
ENSRNOG00000001414	*Serpine1*	2.878		0.421
ENSRNOG00000019996	*Slc16a1*	0.430		0.458
ENSRNOG00000006096	*Slc26a7*	2.423		2.269
ENSRNOG00000015394	*Trpv5*	0.445		2.570
**2 groups: DEX and l-NAME**			**DEX**	**l-NAME**
ENSRNOG00000037206	*Ccdc77*		4.727	0.188
ENSRNOG00000024899	*Cxcl13*		0.160	0.414
ENSRNOG00000029128	*Cyp2d5*		3.162	0.284
ENSRNOG00000021699	*D4A508_RAT*		5.273	0.456
ENSRNOG00000039874	*D4A8E2_RAT*		8.207	0.410
ENSRNOG00000015716	*Gp2*		6.667	2.250
ENSRNOG00000015518	*Rbp4*		5.991	0.463
ENSRNOG00000025670	*Shisa3*		0.101	0.441
ENSRNOG00000030500	*Tcf24*		0.114	1.74 × 10^−5^
ENSRNOG00000020057	*Tex101*		0.061	1.71 × 10^−5^
ENSRNOG00000000768	*Ubd*		39.33	3.45 × 10^−6^

We next used DAVID v6.7 [14] to gain biological insight from our gene lists, identifying enriched Gene Ontology (GO) terms, and find functionally related gene groups. Functional annotation clustering revealed distinct gene networks that were affected by HF. Among them, we found 4 out of the 269 HF-induced DEGs, namely *Aqp2*, *Ptgs1*, *Eph2x*, and *Hba-a2*, were related to regulation of blood pressure (BP) (GO:0008217). Next, we also found five significantly related Kyoto Encyclopedia of Genes and Genomes (KEGG) pathways in the kidney of HF-treated offspring, including arachidonic acid metabolism, PPAR signaling, complement and coagulation cascades, circadian rhythm, retinol metabolism, and fructose and mannose metabolism. These data have been published previously [8]. Next, we found no significant related KEGG pathways in DEX *vs.* control. In l-NAME model, we found 5 out of the 383 DEGs, namely *Apln*, *Guca2b*, *Hmox1*, *Hba-a2*, and *Npy*, were related to regulation of BP (GO:0008217). Moreover, there were nine significantly related KEGG pathways in the kidney of l-NAME-treated offspring, including circadian rhythm, mitogen-activated protein kinases (MAPK) signaling pathway, colorectal cancer, chemokine signaling pathway, pathways in cancer, Wnt signaling pathway, NOD-like receptor signaling pathway, renal cell carcinoma, and prion diseases. Note that RNA-Seq data from l-NAME group, which in part was published previously [13], is included in this study for the sake of comparison.

### 2.3. Gene Expression of Arachidonic Acid Metabolism Pathway

**Figure 3:**
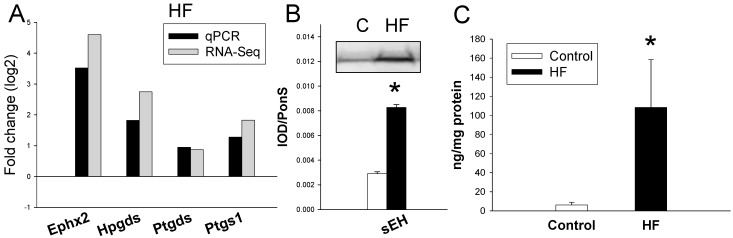

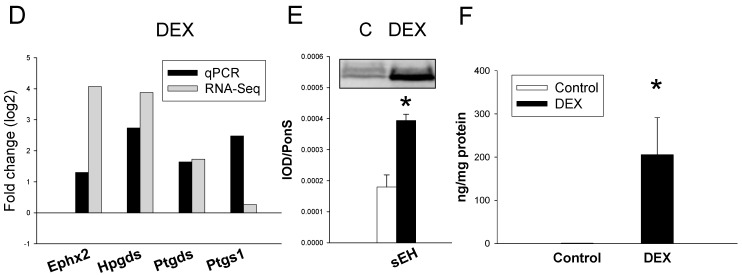
(**A**) Effects of HF on gene expression of arachidonic acid metabolism pathway in the kidney. Confirmatory analysis of kidney gene expression by qPCR in HF *vs.* control. Individual gene expression was determined by qPCR and expressed as fold change in log base 2 *vs.* control and graphed alongside fold change derived from RNA-Seq analysis; (**B**) Representative Western blots showing soluble epoxide hydrolase (SEH) protein (62 kDa) in offspring kidneys at 12 weeks of age. Relative abundance of renal sEH is quantified; (**C**) Effect of HF on renal 14,15-dihydroxyeicosatrienoic acid level; (**D**) Effects of DEX on gene expression of arachidonic acid metabolism pathway in the kidney. Confirmatory analysis of kidney gene expression by qPCR in DEX *vs.* control; (**E**) Representative Western blots showing sEH protein in offspring kidneys at 16 weeks of age; (**F**) Effect of DEX on renal 14,15-dihydroxyeicosatrienoic acid level. * *p* < 0.05 *vs.* control.

Given that there is a significant increase of gene *Ephx2* induced by HF or DEX (Table 1), that arachidonic acid metabolism is a significant related KEGG pathway in the HF model, and that arachidonic acid metabolites play important roles on BP control [15], we analyzed 4 upregulated genes (*Ephx2*, *Hpgds*, *Ptgds*, and *Ptgs1*) in arachidonic acid metabolism pathway. We observed that HF and DEX induced the increased mRNA levels of *Ephx2*, *Hpgds*, *Ptgds*, and *Ptgs1*. The results obtained were in agreement with RNA-seq data (Figure 3A,D). Since *Ephx2* is an identified DEG and is involved in the arachidonic acid pathway, we next analyzed soluble epoxide hydrolase (sEH, encoding *Ephx2* gene) protein level and its activity in the kidney. We found sEH protein level was higher in the HF group compared to control (Figure 3B). Moreover, HF increased renal 14,15-dihydroxyeicosatrienoic acids (DHET) level, a marker representing sEH activity (Figure 3C) [15]. Similarly, renal sEH protein level and 14,15-DHET level were higher in DEX group than those in control (Figure 3E,F). Our results demonstrated HF and DEX similarly affected arachidonic acid metabolism pathway.

## 3. Discussion

This study provides insight into a number of gatekeeper genes and pathways by which different prenatal insults elicit the same phenotype in the adult offspring—programmed hypertension. The key findings are: (1) maternal exposure to DEX, HF, or l-NAME causes increases in BP in adult male offspring; (2) maternal DEX, HF, or l-NAME exposure elicits long-term renal programming by ~0.2%–0.8% differential gene expression in adult offspring kidney; (3) a total of five shared genes (*Bcl6*, *Dmrtc1c*, *Egr1*, *Inmt*, and *Olr1668*) are identified among DEX *vs.* control, HF *vs.* control, and l-NAME *vs.* control group; (4) the identified DEGs, including *Aqp2*, *Ptgs1*, *Eph2x*, *Hba-a2*, *Apln*, *Guca2b*, *Hmox1*, and *Npy*, are related to regulation of BP; and (5) RNA-Seq identified genes in arachidonic acid metabolism are potentially common genes and pathways contributing to programmed hypertension.

Some particular genes and pathways related to programmed hypertension have been studies in different models of developmental programming such as genes for nephrogenesis, oxidative stress, epigenetic regulation, RAS, and sodium transporters [4,5,6,7,8,9,10,11,12,13]. On the other hand, there might be some gatekeeper genes/pathways are common to all prenatal insults [16]. In RNA-Seq dataset, we observed arachidonic acid metabolism is a significant KEGG pathway shared by HF and DEX models. Arachidonic acid is metabolized by cyclooxygenase (COX), lipoxygenase, or cytochrome P450 (CYP) [17]. The epxygenase CYP enzymes generate epoxyeicosatrienoic acids (EETs). EETs can be hydrolyzed by sEH to generate DHETs. Our results demonstrated HF and DEX similarly affected *Ephx2*-sEH-DHET pathway (Figure 3). Given that increased expression/activity of sEH and increased 14,15-DHET level have been associated with hypertension [17,18], our results indicated that programmed hypertension induced by HF and DEX is, at least in part, attributed to sEH and arachidonic acid pathway.

Interestingly, a total of 5 DEGs were identified shared among three different models, including *Bcl6*, *Dmrtc1c*, *Egr1*, *Inmt*, and *Olr1668*. To the best of our knowledge, none of them has shown a direct relationship with hypertension. *Bcl6* gene encodes B-cell CLL/lymphoma 6, which is involved in actin cytoskeleton organization, B cell differentiation, and cell morphogenesis. Although *Bcl6* is not related to BP control, a recent study showing that Bcl6 is involved in glucocorticoid mediated transcription of prostaglandin transporter [19]. Given that prostaglandin is one of arachidonic acid metabolites, our finding suggest *Bcl6* might be involved in arachidonic acid pathway, to elicit programmed hypertension. Next, *Dmrc1c* gene encodes to DMRT-like family C1c1. The *Dmrt* genes encode a large family of transcription factors whose function in sexual development [20]. Nevertheless, their roles in hypertension remain unknown. *Egr1* encodes for early growth response-1, a zinc-finger transcription factor. *Egr1* can be activated by oxidative stress to promote atherosclerosis, diabetes, and pulmonary hypertension [21]. We and others have demonstrated that oxidative stress is involved in the development of hypertension in various programming models [4,5,6,7]. Thus it is likely that *Egr1* is related to oxidative stress-induced programmed hypertension. *Inmt* gene encodes for indolethylamine *N*-methyltransferase (INMT). Bufotenine and *N*,*N*-dimethyltryptamine (DMT) are hallucinogenic dimethylated indolethylamines formed from serotonin and tryptamine by the enzyme INMT [22]. So far, there had been no reports of INMT linking to hypertension. Last, *Olr1668* encodes to olfactory receptor 1668. Olfactory receptors are G protein-coupled receptors that mediate olfactory chemosensation and serve as chemosensors in other tissues. *Olr1668* is one of olfactory receptors expressed in the kidney. Like *Bcl6*, a recent report showing that olfactory receptors are involved in prostaglandin synthesis [23]. This finding further strengthens the potential role of arachidonic acid pathway as a common pathway in the development of programmed hypertension. It is noteworthy that these common genes and pathways need further investigation because of the limited numbers of models used in this study. Moreover, only a few DEGs and KEGG pathways are selected for confirmation; further studies are needed to validate other candidate genes and pathways in our RNA-Seq data.

In addition to sEH (encoded by *Ephx2*), several DEGs identified in the present study are related to regulation of BP (GO: 0008217) including *Aqp2*, *Ptgs1*, *Hba-a2*, *Apln*, *Guca2b*, *Hmox1*, and *Npy*. *Aqp2* gene encodes for aquaporin-2; and renal aquaporin-2 is associated with hypertension due to its ability to maintain water balance [24]. *Ptgs1* gene encodes cyclooxygenase-1 (COX-1). COX-derived prostanoids in the vascular alterations were observed in hypertension [25], which was supported by our RNA-Seq data. *Hba-a2* gene encodes to α1 hemoglobin, which is involved in negative regulation of BP [26]. *Apln* encodes for apelin. Apelin and its receptor are involved in negative regulation of BP [27]. *Guca2b* gene encodes guanylate cyclase activator 2b, which is responsive for NO-mediated cGMP production leading to vasodilatation [28]. *Hmox1* encodes for heme oxygenase 1. It has been implicated in BP regulation [29]. *Npy* encodes the neuropeptide Y. The primary role of *Npy* is a neurotransmitter in the brain and in the autonomic nervous system. It is produced mainly by neurons and serves as a strong vasoconstrictor [30]. However, these identified genes related to BP control are not entirely shared by three different models. They are unable to determine the common underlying mechanisms and explain the commonality among models. Therefore, these candidate proteins encoded by the identified DEGs to be served as gatekeeper genes on hypertension in different programming models require further investigation. In addition, anatomic demarcation, biological functions, and gene expression profiles are different between the kidney cortex and medulla. Given that our previous studies focus on studying renal cortex in programming hypertension [5,6,7,8,9,10,11] and that a greater number of genes are regulated in the kidney cortex than the medulla in hypertensive patients [31], we choose kidney cortex only for RNA-Seq analysis in this study. We cannot tell whether genes and pathways in the kidney medulla appear relevant to programmed hypertension and merit further analyses.

Our results indicated there is a tendency that shared DEGs between HF and DEX are different from those in l-NAME group. The DEGs upregulated by DEX or HF (e.g., *Dmrtc1c*, *Egr1*, and *Olr1668*), are reciprocally downregulated by l-NAME exposure. Next, a half of the DEGs (*Ccdc77*, *Cyp2d5*, *Rbp4*, and *etc.*) shared by DEX and l-NAME group, are upregulated by one but downregulated by the other. Chronic inhibition of NO with l-NAME causes hypertension and preeclampsia in pregnant rodents [32], which are different from those mother rats exposed to DEX and HF. Also, we cannot absolutely rule out the possibility that five genes shared by three models occur in later life is a secondary phenomenon. It is possible that changes in transcriptome expression in adult offspring kidney could be the consequences to renal programming. Since nephrogenesis occurs from late gestation to postnatal week 1–2 in rodents, analyzing transcriptome in the developing kidneys to capture candidate genes and pathways might aid in identifying primary programmed changes in response to different insults. So far there are few, or none, RNA-Seq report from the rat kidney have been reported using our three programming models. The offspring of l-NAME-treated pregnant rats exhibited intrauterine growth retardation (IUGR). We compared the l-NAME-induced DEGs to data from the fetal kidney in an IUGR model [33]. We found epigenetic regulator genes are similarly unchanged in our l-NAME model and in the IUGR model [33], suggesting epigenetic regulators might not be the major route by which l-NAME and IUGR affect the renal programming. Moreover, the DEGs in our prenatal DEX model are completely different from those acute corticosteroid-regulated genes using the microarray approach [34], indicting the underlying mechanisms between two corticosteroid-related models are distinct. Despite all three models exhibit programmed hypertension, there might be different compensatory mechanisms for deficits, leading to differentially regulated genes between diverse programming models. Thereby, a greater understanding of the similarity and difference among a variety of programming models with common phenotype is essential to devise preventative measures and improve therapeutic strategies.

## 4. Experimental Section

### 4.1. Animals

This study was carried out in strict accordance with the recommendations in the Guide for the Care and Use of Laboratory Animals of the National Institutes of Health. The protocol was approved by the Institutional Animal Care and Use Committee of the Kaohsiung Chang Gung Memorial Hospital (Permit Numbers: 2012101601 and 2012022202 and 2011120602). Sprague Dawley rats (10 week-old) were obtained (BioLASCO Taiwan Co., Ltd., Taipei, Taiwan) and maintained in a facility accredited by the Association for Assessment and Accreditation of Laboratory Animal Care International. Male rats were caged with individual females until mating was confirmed.

In the DEX model, dexamethasone (DEX, 0.1 mg/kg body weight) or vehicle was intraperitoneally administered to pregnant SD rats from gestational day 16 to 22 as we reported previously [10]. Male offspring were assigned to control and DEX group. Animal was killed at 16 weeks of age. In the HF model, pregnant SD rats received regular chow or chow supplemented with fructose (60% diet by weight) during the whole period of pregnancy and lactation. Male offspring were assigned to two groups: control and HF [8]. Rats were sacrificed at 12 weeks of age. In the l-NAME model, pregnancy rats receive N^G^-nitro-l-arginine-methyester (l-NAME) administration at 60 mg/kg per day by a subcutaneous osmotic pump (Alza Corporation, Palo Alto, CA, USA) during the whole period of pregnancy [9]. Pregnant rats received continuous infusion of iso-osmotic saline were used as controls. Male offspring was assigned to control and l-NAME group. Male offspring was sacrificed at 12 weeks of age. BP was measured in conscious rats by an indirect tail-cuff method (BP-2000, Visitech Systems, Inc., Apex, NC, USA) [8,9,10]. To ensure accuracy and reproducibility, 1 week prior to the experiment the rats were trained to restraint and tail-cuff inflation. BP measurements were taken between 1300 and 1700 each day. Rats were placed on specimen platform. Their tails were passed through tail cuffs and secured in place with tape. Following a 10-min warm-up period, 10 preliminary cycles of tail-cuff inflation were performed. For each rat, 5 cycles were recorded at each time point. Average of values from three stable measurements was taken. Heparinized blood samples were collected. Kidney was removed, separated into cortex and medulla, snapped frozen, and stored at −80 °C freezer for further analysis. Renal 14,15-DHET level was measured using a commercially available ELISA kit (Detroit R&D Inc., Detroit, MI, USA) following manufacturer’s protocol.

### 4.2. Next-Generation Sequencing and Analysis

Kidney cortex (*n* = 3/group) was isolated for whole-genome RNA next-generation sequencing (RNA-Seq), performed by Welgene Biotech Co., Ltd. (Taipei, Taiwan). Purified RNA was quantified at 260 nm (OD_600_) by using a ND-1000 spectrophotometer (Nanodrop Technology, Wilmington, DE, USA) and analyzed using a Bioanalyzer 2100 (Agilent Technology, Santa Clara, CA, USA) with RNA 6000 LabChip kit (Agilent Technologies). All procedures were performed according to the Illumina protocol (Illumina Inc., San Diego, CA, USA). For all samples, library construction was performed using the TruSeq RNA Sample Prep Kit v2 for ~160 bp (single-end) sequencing and the Solexa platform (Illumina Inc.). The reference genome and gene annotations were retrieved from Ensembl database. The sequence was directly determined by sequencing-by-synthesis technology using the TruSeq SBS Kit (Illumina Inc.). Raw sequences were obtained using the Illumina GA Pipeline software CASAVA v1.8, which was expected to generate 30 million reads per sample. The sequences generated went through a filtering process to obtain qualified reads. ConDeTri [35] was implemented to trim or remove the reads according to the quality score. Qualified reads after filtering low-quality data were analyzed using TopHat/Cufflinks [36] for gene expression estimation. The gene expression level was calculated as fragments per kilobase of transcript per million mapped reads (FPKM). For differential expression analysis, CummeRbund (Illumina Inc.) was employed to perform statistical analyses of gene expression profiles. The cuffdiff tool from the cufflinks package was run to calculate expression changes and associated q values (false discovery rate adjusted *p* values) for each gene between control and DEX, control and HF, as well as control and l-NAME group. The output files of cuffdiff were further annotated by adding gene functional descriptions and GO classifications. GO term enrichment and fold enrichment or depletion for gene lists of significantly up- and downregulated genes in kidney were determined. GO analysis for significant genes and KEGG pathway were analyzed using NIH DAVID Bioinformatics Resources 6.7 [14].

### 4.3. Quantitative Real-Time PCR Analysis

RNA was extracted as previously described procedures [8,9,10,11,12]. Two-step quantitative real-time PCR (qPCR) was conducted using Quantitect SYBR Green PCR Reagents (Qiagen, Valencia, CA, USA) on an iCycler iQ Multi-color Real-Time PCR Detection System (Bio-Rad, Hercules, CA, USA). Four genes involved in arachidonic acid metabolism, including *Ephx2*, *Hpgds*, *Ptgds*, and *Ptgs1* (encodes Cox1), were analyzed. *R18s* was used as a reference in all analyses. Primers were designed using GeneTool Software (Biotools, Edmonton, AB, Canada) (Table 2). All samples were run in duplicate. For the relative quantification of gene expression, the comparative threshold cycle (*C*_t_) method was employed. The averaged *C*_t_ was subtracted from the corresponding averaged *R18s* value for each sample, resulting in Δ*C*_t_. ΔΔ*C*_t_ was achieved by subtracting the average control Δ*C*_t_ value from the average experimental Δ*C*_t_. The fold-increase was established by calculating 2^−ΔΔ*C*t^ for experimental *vs.* reference samples.

**Table 2:** qPCR primers sequences.

Gene	Forward	Reverse
*Bcl6*	5'-CTGAGGGAAGGCAACATCAT-3'	5'-CGGCTGTTCAGGAACTCTTC-3'
*Dmrtc1c*	5'-ACATACAAGTCACGCTGGCA-3'	5'-TTGGCCTGTTTGAGGGGTTT-3'
*Egr1*	5'-CAGGAGTGATGAACGCAAGA-3'	5'-AGCCCGGAGAGGAGTAAGAG-3'
*Inmt*	5'-CAGGAGTGATGAACGCAAGA-3'	5'-AGCCCGGAGAGGAGTAAGAG-3'
*Olr1668*	5'-ACGTGGCTATCTGCAGACCT-3'	5'-CTCCCCACAGGCAGTTTTTA-3'
*R18s*	5'-GCCGCGGTAATTCCAGCTCCA-3'	5'-CCCGCCCGCTCCCAAGATC-3'

*Bcl6* = B-cell CLL/lymphoma 6; *Dmrtc1c* = DMRT-like family C1c1; *Egr1* = early growth response-1; *Inmt* = indolethylamine *N*-methyltransferase; *Olr1668* = olfactory receptor 1668.

### 4.4. Western Blot

Western blot analysis was performed as described previously [5,6]. We used a rabbit anti-rat soluble epoxide hydrolase (sEH) antibody (1:1000, 2 h; Santa Cruz Biotechnology, Santa Cruz, CA, USA). Bands of interest were visualized using SuperSignal West Pico reagent (Pierce; Rockford, IL, USA) and quantified by densitometry as integrated optical density (IOD), factored for Ponceau S red (PonS) staining to correct for any variations in total protein loading. The protein abundance was represented as IOD/PonS.

### 4.5. Statistics

Normally distributed data are given as mean ± S.E.M. For most parameters, statistical analysis was done using student’s *t*-test. A *p*-value <0.05 was considered statistically significant. All analyses were performed using the Statistical Package for the Social Sciences (SPSS) 15.0 statistics software (SPSS Inc., Chicago, IL, USA).

## 5. Conclusions

In conclusion, a variety of prenatal insults elicit programmed hypertension in adult offspring. Using RNA-Seq technology, we identified arachidonic acid pathway might be a gatekeeper pathway contribute to this common phenotype that follow programming insults of HF and DEX. Our RNA-Seq results are of significant to the development of potential interventions in the prevention of programmed hypertension in children exposed antenatal sub-optimal conditions.

## Supplementary Materials

Supplementary materials can be found at http://www.mdpi.com/1422-0067/16/03/4744/s1.
