# Supplementary material for: Transcriptome Analysis in Rat Kidneys: Importance of Genes Involved in Programmed Hypertension
Source: Int J Mol Sci. 2015 Mar 2;16(3):4744–58. doi: 10.3390/ijms16034744 (PMC4394446; doi:10.3390/ijms16034744)
Supplement: Supplementary file 1 [file ijms-16-04744-s001.pdf]

Supplementary Information

Table S1. List of the 58 differentially expressed genes induced by prenatal dexamethasone in 16-week-old rat kidney.

Gene_ID	Gene Symbol	Fold Changes	Log2	*p*-Value
Upregulated: 35 genes				
ENSRNOG00000010950	*Tsga14*	41.21	5.37	0.0387
ENSRNOG00000000768	*Ubd*	39.33	5.30	0.0025
ENSRNOG00000037923	*Dmrtc1c*	38.46	5.27	0.0000
ENSRNOG00000003606	*RGD1561381*	32.69	5.03	0.0001
ENSRNOG00000017286	*HYES_RAT*	16.76	4.07	0.0001
ENSRNOG00000004744	*Fam84b*	15.99	4.00	0.0045
ENSRNOG00000038289	*D3ZH19_RAT*	11.80	3.56	0.0036
ENSRNOG00000021292	-	10.97	3.46	0.0043
ENSRNOG00000003479	*Rnf150*	8.644	3.11	0.0416
ENSRNOG00000034915	*5_8S_rRNA*	8.306	3.05	0.0359
ENSRNOG00000039874	*D4A8E2_RAT*	8.207	3.04	0.0364
ENSRNOG00000032348	*LOC100362957*	6.854	2.78	0.0209
ENSRNOG00000015716	*Gp2*	6.667	2.74	0.0025
ENSRNOG00000032417	*GBRP_RAT*	6.378	2.67	0.0275
ENSRNOG00000042907	*F1M253_RAT*	6.370	2.67	0.0410
ENSRNOG00000019382	*D3ZQI7_RAT*	6.231	2.64	0.0312
ENSRNOG00000015518	*Rbp4*	5.991	2.58	0.0020
ENSRNOG00000042174	*F1M9D9_RAT*	5.824	2.54	0.0131
ENSRNOG00000044121	-	5.802	2.54	0.0272
ENSRNOG00000012531	*Ephb2*	5.554	2.47	0.0253
ENSRNOG00000045400	-	5.356	2.42	0.0361
ENSRNOG00000024243	*Cadm4*	5.348	2.42	0.0416
ENSRNOG00000021699	*D4A508_RAT*	5.273	2.40	0.0485
ENSRNOG00000019131	-	5.262	2.40	0.0040
ENSRNOG00000009947	*Kremen1*	5.025	2.33	0.0498
ENSRNOG00000012428	*Maf*	4.746	2.25	0.0498
ENSRNOG00000037206	*Ccdc77*	4.727	2.24	0.0350
ENSRNOG00000044316	-	4.594	2.20	0.0256
ENSRNOG00000042419	*LOC100361559*	4.576	2.19	0.0462
ENSRNOG00000019661	*Gdf15*	4.500	2.17	0.0480
ENSRNOG00000029889	*Clca4*	4.238	2.08	0.0494
ENSRNOG00000029622	*Olr1668*	4.106	2.04	0.0176
ENSRNOG00000019422	*Egr1*	3.947	1.98	0.0133
ENSRNOG00000029128	*Cyp2d5*	3.162	1.66	0.0346
ENSRNOG00000006325	*Rps21*	3.118	1.64	0.0368

Table S1. *Cont.*

Gene_ID	Gene Symbol	Fold Changes	Log2	*p*-Value
Downregulated: 23 genes				
ENSRNOG00000031706	-	0.317	−1.66	0.0397
ENSRNOG00000042289	*Plcxd2*	0.294	−1.76	0.0364
ENSRNOG00000011250	*Inmt*	0.275	−1.86	0.0216
ENSRNOG00000032274	-	0.272	−1.88	0.0216
ENSRNOG00000007243	*Havcr1*	0.262	−1.94	0.0267
ENSRNOG00000016219	*Vnn1*	0.260	−1.94	0.0142
ENSRNOG00000004147	*D3ZCF8_RAT*	0.255	−1.97	0.0479
ENSRNOG00000003215	*Znf287*	0.253	−1.98	0.0247
ENSRNOG00000001843	*Bcl6*	0.217	−2.21	0.0066
ENSRNOG00000021355	*Car6*	0.209	−2.26	0.0462
ENSRNOG00000034290	*Ccl21*	0.203	−2.30	0.0353
ENSRNOG00000029651	*Rdh2*	0.195	−2.36	0.0138
ENSRNOG00000002947	*Dpt*	0.190	−2.39	0.0125
ENSRNOG00000013717	*F1M089_RAT*	0.184	−2.44	0.0335
ENSRNOG00000008645	*Igfbp3*	0.170	−2.55	0.0044
ENSRNOG00000037374	*D3ZPQ1_RAT*	0.163	−2.62	0.0034
ENSRNOG00000024899	*Cxcl13*	0.160	−2.64	0.0024
ENSRNOG00000012516	*F1M7F8_RAT*	0.126	−2.99	0.0003
ENSRNOG00000018615	*F1M389_RAT*	0.126	−2.99	0.0201
ENSRNOG00000030500	*Tcf24*	0.114	−3.14	0.0287
ENSRNOG00000025670	*Shisa3*	0.101	−3.31	0.0017
ENSRNOG00000020057	*Tex101*	0.061	−4.03	0.0006
ENSRNOG00000033932	-	0.048	−4.38	0.0001

Genes that changed by RPKM > 0.3 and ≧2-fold differences between prenatal DEX-treated offspring *vs.* control at 4 months of age. Genes are sorted by fold changes in descending order.

Table S2. List of the 269 differentially expressed genes induced by high-fructose (HF) intake in 12-week-old rat kidney.

Gene_ID	Gene Symbol	Fold Changes	Log2	*p*-Value
Upregulated: 197 genes				
ENSRNOG00000040819	*SNORD24*	2415	17.88	0.0041
ENSRNOG00000029262	*D3Z9G9_RAT*	40,485	15.31	5.00×10^{-5}
ENSRNOG00000032825	*LOC100365668*	31,177	14.93	5.00×10^{-5}
ENSRNOG00000012871	*LOC100360318*	26,291	14.68	0.0041
ENSRNOG00000001466	*LOC100364896*	16,568	14.02	5.00×10^{-5}
ENSRNOG00000001943	*Retnlg*	10,062	13.30	5.00×10^{-5}
ENSRNOG00000031412	-	6247	12.61	5.00×10^{-5}
ENSRNOG00000006058	*Lypd2*	4303	12.07	5.00×10^{-5}
ENSRNOG00000022022	-	3356	11.71	5.00×10^{-5}
ENSRNOG00000038847	*D3ZRM1_RAT*	2850	11.48	0.0015
ENSRNOG00000038717	*D3ZDK0_RAT*	2702	11.40	0.00705
ENSRNOG00000023965	*F1LUU6_RAT*	1589	10.63	0.001
ENSRNOG00000038963	-	1511	10.56	0.00705
ENSRNOG00000020165	*Eraf*	1466	10.52	0.001
ENSRNOG00000015750	*Wnt7b*	1364	10.41	5.00×10^{-5}
ENSRNOG00000014357	*Gja4*	965	9.91	5.00×10^{-5}
ENSRNOG00000003095	*Fgfbp1*	619	9.27	5.00×10^{-5}
ENSRNOG00000015724	*Gucy2g*	46.59	5.54	5.00×10^{-5}
ENSRNOG00000017286	*Ephx2*	24.34	4.61	5.00×10^{-5}
ENSRNOG00000016393	*UT2_RAT*	17.54	4.13	5.00×10^{-5}
ENSRNOG00000037923	*Dmrtc1c*	12.62	3.66	5.00×10^{-5}
ENSRNOG00000013290	*Nrip3*	12.20	3.61	5.00×10^{-5}
ENSRNOG00000005887	*D4A6I7_RAT*	10.39	3.38	5.00×10^{-5}
ENSRNOG00000013547	*Slc6a12*	10.14	3.34	5.00×10^{-5}
ENSRNOG00000012482	*Ndrg4*	7.679	2.94	5.00×10^{-5}
ENSRNOG00000015071	*Zim1*	7.660	2.94	0.0004
ENSRNOG00000018251	*D3ZAM0_RAT*	7.431	2.89	0.0033
ENSRNOG00000022242	*Cxcl9*	7.343	2.88	0.00045
ENSRNOG00000025654	*D3ZX92_RAT*	7.134	2.83	0.0006
ENSRNOG00000003895	*Rgs1*	6.919	2.79	5.00×10^{-5}
ENSRNOG00000006583	*Hpgds*	6.716	2.75	0.0041
ENSRNOG00000006660	*Ndufa10*	6.402	2.68	0.00015
ENSRNOG00000019536	*Nid67*	6.003	2.59	0.0048
ENSRNOG00000011435	*Osbpl10*	5.997	2.58	0.0079
ENSRNOG00000007740	*Tacstd2*	5.883	2.56	0.00365
ENSRNOG00000005957	*Slc4a7*	5.775	2.53	5.00×10^{-5}
ENSRNOG00000001843	*Bcl6*	5.401	2.43	5.00×10^{-5}
ENSRNOG00000021870	*Slco4a1*	5.371	2.43	0.0082
ENSRNOG00000012989	*Serinc2*	5.326	2.41	0.00095
ENSRNOG00000016043	*Aqp4*	5.282	2.40	5.00×10^{-5}

Table S2. *Cont.*

Gene_ID	Gene Symbol	Fold Changes	Log2	*p*-Value
ENSRNOG00000019564	*Sptbn2*	5.252	2.39	5.00×10^{-5}
ENSRNOG00000028888	*Serpina10*	5.211	2.38	0.005
ENSRNOG00000016752	*Crispld2*	4.900	2.29	0.00105
ENSRNOG00000042033	-	4.868	2.28	0.00265
ENSRNOG00000017882	*RGD1560691*	4.705	2.23	0.0092
ENSRNOG00000029622	*Olr1668*	4.562	2.19	5.00×10^{-5}
ENSRNOG00000015085	*D4A0F0_RAT*	4.552	2.19	0.0022
ENSRNOG00000011971	*C1s*	4.531	2.18	5.00×10^{-5}
ENSRNOG00000021027	*Dbp*	4.491	2.17	5.00×10^{-5}
ENSRNOG00000011631	*Fst*	4.452	2.15	0.00015
ENSRNOG00000003972	*Tshr*	4.418	2.14	0.0003
ENSRNOG00000032495	*Olr1411*	4.409	2.14	0.00155
ENSRNOG00000003745	*Atf3*	4.408	2.14	0.00245
ENSRNOG00000014578	*Fxyd4*	4.345	2.12	5.00×10^{-5}
ENSRNOG00000021357	*Slfn3*	4.289	2.10	0.00075
ENSRNOG00000012460	*Cntf*	4.067	2.02	0.00145
ENSRNOG00000008282	*Elf5*	4.036	2.01	0.0085
ENSRNOG00000043451	*LOC100359743*	3.966	1.99	5.00×10^{-5}
ENSRNOG00000001312	*Pdgfa*	3.929	1.97	5.00×10^{-5}
ENSRNOG00000009513	*Akr1b1*	3.905	1.97	0.0001
ENSRNOG00000023148	*COBA1_RAT*	3.887	1.96	0.00115
ENSRNOG00000010805	*Fabp4*	3.819	1.93	5.00×10^{-5}
ENSRNOG00000026904	*NCKX1_RAT*	3.778	1.92	0.00155
ENSRNOG00000003694	*Prox1*	3.737	1.90	0.00315
ENSRNOG00000017414	*Irf7*	3.684	1.88	0.0003
ENSRNOG00000033488	*Q6QI27_RAT*	3.678	1.88	0.00035
ENSRNOG00000013872	*P2ry14*	3.641	1.86	0.0064
ENSRNOG00000021234	*D3ZVP9_RAT*	3.637	1.86	0.0001
ENSRNOG00000003616	*Grem2*	3.557	1.83	0.00355
ENSRNOG00000007415	*Ptgs1*	3.547	1.83	0.0015
ENSRNOG00000018187	*Racgap1*	3.481	1.80	0.00955
ENSRNOG00000018911	*Pfkfb3*	3.466	1.79	0.00095
ENSRNOG00000001143	*Cit*	3.406	1.77	0.00245
ENSRNOG00000006154	*Pde1a*	3.307	1.73	5.00×10^{-5}
ENSRNOG00000012863	*LOC100363748*	3.294	1.72	0.00055
ENSRNOG00000000906	*RGD1307396*	3.281	1.71	0.00435
ENSRNOG00000015410	*Aspn*	3.267	1.71	0.0001
ENSRNOG00000032378	*LOC100365885*	3.190	1.67	0.0035
ENSRNOG00000003120	*Prelp*	3.168	1.66	0.0013
ENSRNOG00000017087	*Man1c1*	3.149	1.66	5.00×10^{-5}
ENSRNOG00000000036	*Klhdc8a*	3.135	1.65	0.00105
ENSRNOG00000014585	*Ccdc11*	3.134	1.65	0.0007
ENSRNOG00000016696	*Angpt2*	3.103	1.63	0.001

Table S2. *Cont.*

Gene_ID	Gene Symbol	Fold Changes	Log2	*p*-Value
ENSRNOG00000012294	*Heph*	3.102	1.63	0.00065
ENSRNOG00000001688	*Sim2*	3.101	1.63	0.0002
ENSRNOG00000030021	*Ccl6*	3.081	1.62	0.0009
ENSRNOG00000002930	*Ppl*	3.057	1.61	0.0001
ENSRNOG00000017803	*Apbb1ip*	3.054	1.61	0.00335
ENSRNOG00000009431	*D3Z881_RAT*	3.051	1.61	5.00×10^{-5}
ENSRNOG00000034303	*Spon1*	3.007	1.59	5.00×10^{-5}
ENSRNOG00000039096	*F1M0C9_RAT*	2.985	1.58	0.0001
ENSRNOG00000011019	*Faah*	2.983	1.58	0.0034
ENSRNOG00000001145	*Ccdc64*	2.963	1.57	0.00015
ENSRNOG00000019206	*Nupr1*	2.962	1.57	5.00×10^{-5}
ENSRNOG00000032240	*Gbp5*	2.952	1.56	0.0013
ENSRNOG00000005772	*Ptplad2*	2.935	1.55	0.0058
ENSRNOG00000006860	*Itk*	2.923	1.55	0.00295
ENSRNOG00000000296	*Aqp6*	2.923	1.55	5.00×10^{-5}
ENSRNOG00000004517	*Igf1*	2.921	1.55	5.00×10^{-5}
ENSRNOG00000008015	*Fos*	2.897	1.53	0.00255
ENSRNOG00000019422	*Egr1*	2.885	1.53	5.00×10^{-5}
ENSRNOG00000019741	*Isyna1*	2.883	1.53	0.00325
ENSRNOG00000007202	*Sema3d*	2.881	1.53	5.00×10^{-5}
ENSRNOG00000001414	*Serpine1*	2.878	1.52	0.0012
ENSRNOG00000011796	*C1r*	2.843	1.51	0.0001
ENSRNOG00000013179	*Tinagl1*	2.839	1.51	0.0009
ENSRNOG00000021478	*Tpd52l1*	2.831	1.50	0.00425
ENSRNOG00000000297	*Aqp2*	2.828	1.50	5.00×10^{-5}
ENSRNOG00000019673	*Zfp36*	2.812	1.49	5.00×10^{-5}
ENSRNOG00000026053	*GREM1_RAT*	2.798	1.48	5.00×10^{-5}
ENSRNOG00000013825	*Rap1gap*	2.793	1.48	0.00045
ENSRNOG00000007687	*Sema7a*	2.790	1.48	0.0018
ENSRNOG00000015346	*Obsl1*	2.761	1.47	0.0084
ENSRNOG00000017424	*Chrna2*	2.755	1.46	0.00515
ENSRNOG00000011526	*Pcsk6*	2.749	1.46	0.00045
ENSRNOG00000009005	*Slco2a1*	2.736	1.45	0.0014
ENSRNOG00000013090	*Gadd45g*	2.732	1.45	0.0016
ENSRNOG00000021088	*Tmod4*	2.715	1.44	0.0038
ENSRNOG00000001959	*Mx1*	2.708	1.44	0.0012
ENSRNOG00000020951	*Slc4a1*	2.704	1.44	5.00×10^{-5}
ENSRNOG00000011913	*Cp*	2.698	1.43	0.0001
ENSRNOG00000015867	*Chst9*	2.687	1.43	0.00055
ENSRNOG00000002579	*Parm1*	2.667	1.42	5.00×10^{-5}
ENSRNOG00000001158	*Abcg1*	2.659	1.41	0.00265
ENSRNOG00000007830	*Apold1*	2.658	1.41	0.01
ENSRNOG00000001956	*Dzip3*	2.631	1.40	0.00265

Table S2. *Cont.*

Gene_ID	Gene Symbol	Fold Changes	Log2	*p*-Value
ENSRNOG00000013886	*Fyb*	2.629	1.39	0.00525
ENSRNOG00000009797	*Aqp3*	2.625	1.39	5.00×10^{-5}
ENSRNOG00000033465	*Hbb*	2.608	1.38	0.00315
ENSRNOG00000017021	*E9PSI9_RAT*	2.607	1.38	5.00×10^{-5}
ENSRNOG00000004498	*Scin*	2.607	1.38	0.0001
ENSRNOG00000007679	*Cyth4*	2.606	1.38	0.00375
ENSRNOG00000006774	*Ccr5*	2.602	1.38	0.0049
ENSRNOG00000022839	*Ifit3*	2.562	1.36	0.00805
ENSRNOG00000042704	*F1LN80_RAT*	2.556	1.35	0.00025
ENSRNOG00000028278	*RGD1562655*	2.538	1.34	0.00335
ENSRNOG00000009345	*Ugt8*	2.530	1.34	5.00×10^{-5}
ENSRNOG00000030183	*Plod2*	2.520	1.33	5.00×10^{-5}
ENSRNOG00000023465	*LOC500300*	2.513	1.33	0.0058
ENSRNOG00000010165	*D3ZYQ3_RAT*	2.492	1.32	0.0097
ENSRNOG00000014322	*LOC313672*	2.487	1.31	5.00×10^{-5}
ENSRNOG00000012181	*Lpl*	2.470	1.30	5.00×10^{-5}
ENSRNOG00000017440	*Bgn*	2.466	1.30	0.00065
ENSRNOG00000026435	*Arid3a*	2.456	1.30	0.00705
ENSRNOG00000006096	*Slc26a7*	2.423	1.28	0.00575
ENSRNOG00000003687	*Rgs2*	2.416	1.27	0.0026
ENSRNOG00000007484	*Ehf*	2.398	1.26	5.00×10^{-5}
ENSRNOG00000012490	*Amph*	2.398	1.26	0.0045
ENSRNOG00000006761	*Sh3gl2*	2.392	1.26	0.01
ENSRNOG00000003977	*Dusp1*	2.379	1.25	0.00015
ENSRNOG00000016301	*Dmrt2*	2.361	1.24	0.00495
ENSRNOG00000030930	*Samsn1*	2.329	1.22	0.00375
ENSRNOG00000006557	*D3Z8C3_RAT*	2.326	1.22	5.00×10^{-5}
ENSRNOG00000002010	*LOC100360017*	2.320	1.21	0.0001
ENSRNOG00000000577	*Ddit4*	2.319	1.21	0.00425
ENSRNOG00000020406	*Tmed6*	2.305	1.20	0.00235
ENSRNOG00000025411	*F2r*	2.287	1.19	0.0006
ENSRNOG00000007367	*4-Sep*	2.284	1.19	5.00×10^{-5}
ENSRNOG00000028404	*Ppp1r1b*	2.278	1.19	0.0002
ENSRNOG00000017163	*Pfkp*	2.271	1.18	0.00035
ENSRNOG00000019014	*Ndst1*	2.264	1.18	0.00015
ENSRNOG00000029178	*Abcc5*	2.257	1.17	0.0063
ENSRNOG00000012531	*Ephb2*	2.254	1.17	0.0086
ENSRNOG00000003300	*Btg2*	2.253	1.17	0.00045
ENSRNOG00000017283	*Kcnt1*	2.248	1.17	0.00495
ENSRNOG00000015911	*F1MAD0_RAT*	2.221	1.15	0.00045
ENSRNOG00000003217	*Lgals3bp*	2.214	1.15	0.00485
ENSRNOG00000003242	*Gulp1*	2.203	1.14	5.00×10^{-5}
ENSRNOG00000004084	*Fam84a*	2.192	1.13	0.00055

Table S2. *Cont.*

Gene_ID	Gene Symbol	Fold Changes	Log2	*p*-Value
ENSRNOG00000004411	*Tspan8*	2.192	1.13	0.0006
ENSRNOG00000003284	*Epn3*	2.191	1.13	0.00585
ENSRNOG00000005046	*Tspan13*	2.187	1.13	0.00135
ENSRNOG00000013934	*St5*	2.174	1.12	0.0073
ENSRNOG00000005367	*Slc12a1*	2.156	1.11	0.001
ENSRNOG00000009329	*Nr1d1*	2.154	1.11	0.0018
ENSRNOG00000043249	*B0BNK5_RAT*	2.148	1.10	0.00885
ENSRNOG00000015024	*E9PT54_RAT*	2.144	1.10	0.0009
ENSRNOG00000005903	*St14*	2.140	1.10	0.0021
ENSRNOG00000013415	*Ptpn18*	2.138	1.10	0.0096
ENSRNOG00000012516	*F1M7F8_RAT*	2.135	1.09	0.00035
ENSRNOG00000001963	*Mx2*	2.133	1.09	0.00155
ENSRNOG00000017854	*Ucp2*	2.125	1.09	0.0079
ENSRNOG00000010389	*Ndrg2*	2.115	1.08	0.0006
ENSRNOG00000025230	*Ranbp3l*	2.090	1.06	0.00015
ENSRNOG00000039496	*Plp2*	2.075	1.05	0.0094
ENSRNOG00000007478	*Cry2*	2.074	1.05	0.00825
ENSRNOG00000019937	*Kcnk1*	2.059	1.04	0.0061
ENSRNOG00000006646	*Ecop*	2.057	1.04	0.00185
ENSRNOG00000019316	*Sh3bp4*	2.053	1.04	0.0082
ENSRNOG00000018414	*Csf1r*	2.046	1.03	0.00695
ENSRNOG00000010799	*Ccrn4l*	2.043	1.03	0.00335
ENSRNOG00000011886	*MYO1E_RAT*	2.040	1.03	0.0012
ENSRNOG00000002746	*Fstl1*	2.036	1.03	0.0044
ENSRNOG00000017874	*Cd53*	2.014	1.01	0.0013
ENSRNOG00000002815	*F5*	2.012	1.01	0.00055
ENSRNOG00000020254	*Per2*	2.011	1.01	0.00655
ENSRNOG00000029886	*Hba-a2*	2.009	1.01	0.0044
Downregulated: 72 genes				
ENSRNOG00000005447	*RGD1311564*	0.494	−1.02	0.00755
ENSRNOG00000001607	*Adamts1*	0.494	−1.02	0.0037
ENSRNOG00000022609	*Mrps10*	0.486	−1.04	0.00205
ENSRNOG00000013410	*Spink1*	0.484	−1.05	0.0002
ENSRNOG00000030287	*Acaa1a*	0.479	−1.06	0.00105
ENSRNOG00000022141	*CTSL2_RAT*	0.475	−1.07	0.00505
ENSRNOG00000027433	*Akr1b10*	0.474	−1.08	0.0005
ENSRNOG00000004306	*Zbtb39*	0.473	−1.08	0.00335
ENSRNOG00000030154	*Cyp4a2*	0.470	−1.09	0.0013
ENSRNOG00000003108	*Acbd4*	0.468	−1.09	0.00095
ENSRNOG00000036622	*RGD1564894*	0.465	−1.11	0.00705
ENSRNOG00000009086	*Apcs*	0.463	−1.11	5.00×10^{-5}
ENSRNOG00000028288	*Clrn3*	0.456	−1.13	0.0015
ENSRNOG00000007456	*Calb1*	0.454	−1.14	5.00×10^{-5}

Table S2. *Cont.*

Gene_ID	Gene Symbol	Fold Changes	Log2	*p*-Value
ENSRNOG00000005660	*Fam110c*	0.450	−1.15	0.00025
ENSRNOG00000013464	*Spink3*	0.449	−1.16	5.00×10^{-5}
ENSRNOG00000015394	*Trpv5*	0.445	−1.17	0.00595
ENSRNOG00000039350	*Gng13*	0.443	−1.18	0.0007
ENSRNOG00000015406	*Pgm5*	0.438	−1.19	0.00905
ENSRNOG00000032942	*D3ZB63_RAT*	0.437	−1.19	0.00285
ENSRNOG00000023861	*AP180_RAT*	0.436	−1.20	0.00965
ENSRNOG00000013074	*Wt1*	0.434	−1.20	0.00385
ENSRNOG00000019181	*Synpo*	0.433	−1.21	0.0007
ENSRNOG00000040195	*F1LZT0_RAT*	0.432	−1.21	5.00×10^{-5}
ENSRNOG00000019996	*Slc16a1*	0.430	−1.22	0.0001
ENSRNOG00000010999	*K1731_RAT*	0.428	−1.23	0.00215
ENSRNOG00000023338	*Tspan2*	0.421	−1.25	0.002
ENSRNOG00000010421	*Wdr91*	0.415	−1.27	0.00025
ENSRNOG00000026848	*LOC499279*	0.413	−1.28	0.0033
ENSRNOG00000009465	*Sfrp2*	0.411	−1.28	0.00165
ENSRNOG00000012458	*Cyp2e1*	0.409	−1.29	5.00×10^{-5}
ENSRNOG00000015020	*Idh1*	0.404	−1.31	5.00×10^{-5}
ENSRNOG00000018681	*NEST_RAT*	0.403	−1.31	5.00×10^{-5}
ENSRNOG00000012772	*Nqo1*	0.394	−1.34	0.00435
ENSRNOG00000009862	*Olfm1*	0.391	−1.35	0.00515
ENSRNOG00000014090	*Retsat*	0.377	−1.41	5.00×10^{-5}
ENSRNOG00000018693	*Asgr1*	0.367	−1.45	0.0042
ENSRNOG00000001980	*Ugt2b36*	0.365	−1.45	5.00×10^{-5}
ENSRNOG00000003251	*B3galt2*	0.363	−1.46	0.0084
ENSRNOG00000017546	*Mylk3*	0.352	−1.51	0.002
ENSRNOG00000014798	*RGD1309540*	0.346	−1.53	0.00015
ENSRNOG00000013950	*Aadac*	0.345	−1.54	0.00045
ENSRNOG00000011585	*Fat3*	0.337	−1.57	0.0034
ENSRNOG00000000368	*GRIK2_RAT*	0.333	−1.59	0.00365
ENSRNOG00000037853	*Rarres1*	0.329	−1.60	5.00×10^{-5}
ENSRNOG00000006611	*NOSTN_RAT*	0.329	−1.60	0.0001
ENSRNOG00000020272	*F1MAB2_RAT*	0.327	−1.61	0.0081
ENSRNOG00000019120	*Hmgcs2*	0.318	−1.65	0.00035
ENSRNOG00000001765	*Ostalpha*	0.300	−1.74	0.002
ENSRNOG00000004101	*Mosc1*	0.296	−1.76	0.0002
ENSRNOG00000032857	*Klks3*	0.284	−1.81	0.0008
ENSRNOG00000011250	*Inmt*	0.283	−1.82	5.00×10^{-5}
ENSRNOG00000042225	*F1M0H7_RAT*	0.278	−1.85	0.00195
ENSRNOG00000014071	*LOC100362121*	0.267	−1.90	0.00545
ENSRNOG00000017560	*Mdk*	0.266	−1.91	0.0058
ENSRNOG00000005542	*Apob*	0.254	−1.98	5.00×10^{-5}
ENSRNOG00000038455	*Gpx2*	0.241	−2.05	0.00075

Table S2. *Cont.*

Gene_ID	Gene Symbol	Fold Changes	Log2	*p*-Value
ENSRNOG00000029651	*Rdh2*	0.228	−2.13	5.00×10^{-5}
ENSRNOG00000038058	*F1LYF8_RAT*	0.209	−2.26	0.0002
ENSRNOG00000030492	*LOC100364577*	0.199	−2.33	5.00×10^{-5}
ENSRNOG00000009734	*Akr1b8*	0.032	−4.97	0.00165
ENSRNOG00000012067	*Fam111a*	0.018	−5.80	5.00×10^{-5}
ENSRNOG00000024580	*Mamstr*	0.001	−9.50	0.0063
ENSRNOG00000024626	-	9.49×10^{-4}	−10.04	0.00295
ENSRNOG00000032926	*Crygb*	8.79×10^{-4}	−10.15	0.001
ENSRNOG00000039825	*Rcc1*	4.67×10^{-4}	−11.06	0.00295
ENSRNOG00000029919	*F1M7Y9_RAT*	4.44×10^{-4}	−11.14	5.00×10^{-5}
ENSRNOG00000017412	*D4A6V3_RAT*	1.54×10^{-4}	−12.66	5.00×10^{-5}
ENSRNOG00000015156	*Gal*	6.01×10^{-5}	−14.02	5.00×10^{-5}
ENSRNOG00000040692	*SNORD59*	1.64×10^{-5}	−15.90	0.0041
ENSRNOG00000043823	-	7.22×10^{-6}	−17.08	0.00955
ENSRNOG00000041331	-	3.59×10^{-6}	−18.09	0.0001
ENSRNOG00000005447	*RGD1311564*	0.494	−1.02	0.00755
ENSRNOG00000001607	*Adamts1*	0.494	−1.02	0.0037
ENSRNOG00000022609	*Mrps10*	0.486	−1.04	0.00205
ENSRNOG00000013410	*Spink1*	0.484	−1.05	0.0002
ENSRNOG00000030287	*Acaa1a*	0.479	−1.06	0.00105
ENSRNOG00000022141	*CTSL2_RAT*	0.475	−1.07	0.00505
ENSRNOG00000027433	*Akr1b10*	0.474	−1.08	0.0005
ENSRNOG00000004306	*Zbtb39*	0.473	−1.08	0.00335
ENSRNOG00000030154	*Cyp4a2*	0.470	−1.09	0.0013
ENSRNOG00000003108	*Acbd4*	0.468	−1.09	0.00095
ENSRNOG00000036622	*RGD1564894*	0.465	−1.11	0.00705
ENSRNOG00000009086	*Apcs*	0.463	−1.11	5.00×10^{-5}
ENSRNOG00000028288	*Clrn3*	0.456	−1.13	0.0015
ENSRNOG00000007456	*Calb1*	0.454	−1.14	5.00×10^{-5}
ENSRNOG00000005660	*Fam110c*	0.450	−1.15	0.00025
ENSRNOG00000024580	*Mamstr*	0.001	−9.50	0.0063
ENSRNOG00000024626	-	9.49×10^{-4}	−10.04	0.00295
ENSRNOG00000032926	*Crygb*	8.79×10^{-4}	−10.15	0.001
ENSRNOG00000039825	*Rcc1*	4.67×10^{-4}	−11.06	0.00295
ENSRNOG00000029919	*F1M7Y9_RAT*	4.44×10^{-4}	−11.14	5.00×10^{-5}
ENSRNOG00000017412	*D4A6V3_RAT*	1.54×10^{-4}	−12.66	5.00×10^{-5}
ENSRNOG00000015156	*Gal*	6.01×10^{-5}	−14.02	5.00×10^{-5}
ENSRNOG00000040692	*SNORD59*	1.64×10^{-5}	−15.90	0.0041
ENSRNOG00000043823	-	7.22×10^{-6}	−17.08	0.00955
ENSRNOG00000041331	-	3.59×10^{-6}	−18.09	0.0001

Genes that changed by RPKM > 0.3 and ≧2-fold differences between HF-treated offspring *vs*. control at 3 months of age. Genes are sorted by fold changes in descending order.

Table S3. List of the 383 differentially expressed genes induced by L-NAME in 12-week-old rat kidney.

Gene_ID	Gene Symbol	Fold Changes	Log2	*p*-Value
Upregulated: 198 genes				
ENSRNOG00000043638	-	323,734	18.30	0.02325
ENSRNOG00000040314	*F1LXH0_RAT*	301,093	18.20	0.01665
ENSRNOG00000012871	*LOC100360318*	127,959	16.97	0.02325
ENSRNOG00000035040	*SNORA31*	89,333	16.45	0.01615
ENSRNOG00000034029	*F1M1X4_RAT*	51,286	15.65	0.02325
ENSRNOG00000016278	*Ccl17*	23,560	14.52	0.02325
ENSRNOG00000038599	*D3ZCN0_RAT*	15,499	13.92	0.00375
ENSRNOG00000012103	*LOC100361806*	15,395	13.91	0.00005
ENSRNOG00000038906	*Hist1h2ail*	14,661	13.84	0.0094
ENSRNOG00000031914	*F1M5M6_RAT*	13,355	13.71	0.02325
ENSRNOG00000018426	*Apoc1*	12,416	13.60	0.0094
ENSRNOG00000039810	*D4A199_RAT*	11,913	13.54	0.0094
ENSRNOG00000042234	*D3ZDW4_RAT*	10,601	13.37	0.0094
ENSRNOG00000039439	*F1M772_RAT*	8667	13.08	0.005
ENSRNOG00000030428	*LOC100363282*	6444	12.65	0.01135
ENSRNOG00000029844	*Akr1c2*	1072	10.07	0.02415
ENSRNOG00000033517	*LOC100360791*	474	8.89	0.01715
ENSRNOG00000019500	*Cyp1a1*	14.50	3.86	0.0025
ENSRNOG00000015724	*Gucy2g*	10.45	3.39	0.00405
ENSRNOG00000028913	*LOC689287*	10.21	3.35	0.01805
ENSRNOG00000042717	*RGD1566380*	8.946	3.16	0.04695
ENSRNOG00000003616	*Grem2*	8.133	3.02	0.0124
ENSRNOG00000008337	*Gjd2*	7.710	2.95	0.00175
ENSRNOG00000014510	*F1M0Q8_RAT*	7.076	2.82	0.0259
ENSRNOG00000032327	*Pdia5*	6.886	2.78	0.00005
ENSRNOG00000024399	*CLM8_RAT*	6.587	2.72	0.03375
ENSRNOG00000021027	*Dbp*	6.501	2.70	0.00005
ENSRNOG00000009660	*Enpp6*	6.467	2.69	0.00005
ENSRNOG00000036802	*Snhg11*	6.309	2.66	0.01735
ENSRNOG00000032315	-	6.085	2.61	0.0095
ENSRNOG00000018241	*Ank1*	5.436	2.44	0.03735
ENSRNOG00000027001	*ROA3_RAT*	4.486	2.17	0.00005
ENSRNOG00000040266	*Cdkl4*	4.458	2.16	0.04235
ENSRNOG00000025639	*Slc39a12*	4.344	2.12	0.01035
ENSRNOG00000018143	*Hpdl*	4.235	2.08	0.00705
ENSRNOG00000011424	*Cldn23*	4.175	2.06	0.0433
ENSRNOG00000019120	*Hmgcs2*	4.087	2.03	0.00005
ENSRNOG00000001843	*Bcl6*	4.065	2.02	0.00005
ENSRNOG00000038455	*Gpx2*	3.994	2.00	0.00005
ENSRNOG00000022588	*D3ZPY8_RAT*	3.933	1.98	0.00005
ENSRNOG00000033736	*RGD1565168*	3.848	1.94	0.02815

Table S3. *Cont.*

Gene_ID	Gene Symbol	Fold Changes	Log2	*p*-Value
ENSRNOG00000036585	*F1LN76_RAT*	3.840	1.94	0.0075
ENSRNOG00000038331	*Akr1c1*	3.751	1.91	0.00005
ENSRNOG00000002238	*Sema5b*	3.536	1.82	0.00265
ENSRNOG00000033845	*RGD1565088*	3.532	1.82	0.0001
ENSRNOG00000027811	*Lilrb4*	3.519	1.81	0.0005
ENSRNOG00000033260	*Klra5*	3.485	1.80	0.0056
ENSRNOG00000020953	*Ms4a7*	3.443	1.78	0.00545
ENSRNOG00000011578	*RGD1305928*	3.390	1.76	0.02965
ENSRNOG00000036900	*F1M795_RAT*	3.366	1.75	0.0031
ENSRNOG00000010045	*Clecsf6*	3.336	1.74	0.00105
ENSRNOG00000002418	*Tgfb2*	3.330	1.74	0.00005
ENSRNOG00000031167	*Q7TP44_RAT*	3.311	1.73	0.0475
ENSRNOG00000009329	*Nr1d1*	3.297	1.72	0.0027
ENSRNOG00000016248	*Sox18*	3.287	1.72	0.00045
ENSRNOG00000040242	*Epm2a*	3.283	1.72	0.00035
ENSRNOG00000012779	*Msr1*	3.280	1.71	0.00025
ENSRNOG00000002434	*Tmem100*	3.274	1.71	0.0155
ENSRNOG00000031004	*Cyp2j4*	3.265	1.71	0.00005
ENSRNOG00000004557	*Znf763*	3.233	1.69	0.00005
ENSRNOG00000023657	*RGD1565690*	3.224	1.69	0.00095
ENSRNOG00000029709	*F1LYB1_RAT*	3.217	1.69	0.0031
ENSRNOG00000013872	*P2ry14*	3.208	1.68	0.0044
ENSRNOG00000031024	*LOC100360575*	3.201	1.68	0.03295
ENSRNOG00000031785	*Krt76*	3.173	1.67	0.0337
ENSRNOG00000003648	*Cldn6*	3.156	1.66	0.0001
ENSRNOG00000033699	*D3ZG59_RAT*	3.155	1.66	0.00115
ENSRNOG00000011581	*F1M8R4_RAT*	3.146	1.65	0.006
ENSRNOG00000004226	*Irak3*	3.050	1.61	0.0241
ENSRNOG00000037118	*LOC100363228*	3.041	1.60	0.02535
ENSRNOG00000022431	*LOC100125361*	3.017	1.59	0.001
ENSRNOG00000020415	*Ramp2*	2.996	1.58	0.00025
ENSRNOG00000032495	*Olr1411*	2.968	1.57	0.0076
ENSRNOG00000015588	*Nol3*	2.954	1.56	0.0222
ENSRNOG00000011718	*C1rl*	2.885	1.53	0.0072
ENSRNOG00000008534	*B4F7B7_RAT*	2.871	1.52	0.0003
ENSRNOG00000006715	*Ccr1*	2.866	1.52	0.01225
ENSRNOG00000003096	*D4A974_RAT*	2.859	1.52	0.0469
ENSRNOG00000027350	*Tns4*	2.853	1.51	0.0103
ENSRNOG00000038099	-	2.821	1.50	0.033
ENSRNOG00000010999	*K1731_RAT*	2.801	1.49	0.00005
ENSRNOG00000006660	*Ndufa10*	2.797	1.48	0.00005
ENSRNOG00000012801	*Gsto2*	2.791	1.48	0.01745
ENSRNOG00000018413	*Per3*	2.789	1.48	0.0003

Table S3. *Cont.*

Gene_ID	Gene Symbol	Fold Changes	Log2	*p*-Value
ENSRNOG00000012435	*D3ZSM0_RAT*	2.760	1.46	0.0088
ENSRNOG00000022934	*LOC100359960*	2.760	1.46	0.00255
ENSRNOG00000011187	*F1M3V8_RAT*	2.726	1.45	0.0067
ENSRNOG00000031454	*F1LVZ0_RAT*	2.721	1.44	0.023
ENSRNOG00000043234	*Klrd1*	2.709	1.44	0.0046
ENSRNOG00000009636	*Scrn1*	2.702	1.43	0.00075
ENSRNOG00000007545	*Angptl4*	2.696	1.43	0.015
ENSRNOG00000033528	*Tll1*	2.607	1.38	0.0007
ENSRNOG00000003606	*RGD1561381*	2.596	1.38	0.00005
ENSRNOG00000039759	*Gpr34*	2.581	1.37	0.04335
ENSRNOG00000029078	*Nsbp1_predicted*	2.547	1.35	0.0241
ENSRNOG00000025654	*D3ZX92_RAT*	2.540	1.34	0.0092
ENSRNOG00000032158	*F1M6H3_RAT*	2.533	1.34	0.0019
ENSRNOG00000030813	*Dbi*	2.522	1.33	0.00005
ENSRNOG00000029145	-	2.514	1.33	0.00005
ENSRNOG00000002365	*Itm2a*	2.489	1.32	0.01
ENSRNOG00000032274	-	2.482	1.31	0.00005
ENSRNOG00000018615	*F1M389_RAT*	2.482	1.31	0.0101
ENSRNOG00000012634	*Fbxo10*	2.481	1.31	0.0245
ENSRNOG00000028845	*Ebf1*	2.477	1.31	0.02795
ENSRNOG00000032112	-	2.471	1.31	0.00005
ENSRNOG00000033625	*D3ZZN4_RAT*	2.462	1.30	0.04635
ENSRNOG00000038319	*Akr1c19*	2.446	1.29	0.00005
ENSRNOG00000007811	*D3ZLE4_RAT*	2.440	1.29	0.04705
ENSRNOG00000001369	*Oas1a*	2.438	1.29	0.0108
ENSRNOG00000003170	*Nlrp3*	2.438	1.29	0.00645
ENSRNOG00000033932	-	2.436	1.28	0.00025
ENSRNOG00000005868	*Ttc21b*	2.421	1.28	0.00035
ENSRNOG00000022988	*LOC689876*	2.421	1.28	0.00005
ENSRNOG00000005352	*Elf4*	2.421	1.28	0.03135
ENSRNOG00000008979	*Guca2b*	2.406	1.27	0.0155
ENSRNOG00000004063	*RGD1309926*	2.404	1.27	0.00455
ENSRNOG00000042592	*Rgs10*	2.404	1.27	0.00025
ENSRNOG00000033660	*Slc22a13*	2.394	1.26	0.00215
ENSRNOG00000012061	*KPCB_RAT*	2.389	1.26	0.0034
ENSRNOG00000018187	*Racgap1*	2.387	1.26	0.0147
ENSRNOG00000004899	*Kcns3*	2.383	1.25	0.00825
ENSRNOG00000010635	*Igfbp4*	2.374	1.25	0.00005
ENSRNOG00000002204	*Ccdc158*	2.372	1.25	0.02645
ENSRNOG00000007628	*Ptp4a3*	2.371	1.25	0.0164
ENSRNOG00000030021	*Ccl6*	2.354	1.24	0.0001
ENSRNOG00000027808	*Lilra5*	2.352	1.23	0.0004
ENSRNOG00000013521	*Dhfr*	2.350	1.23	0.00175

Table S3. *Cont.*

Gene_ID	Gene Symbol	Fold Changes	Log2	*p*-Value
ENSRNOG00000018797	*Myrip*	2.349	1.23	0.0275
ENSRNOG00000030962	*RGD1564937*	2.342	1.23	0.0044
ENSRNOG00000006774	*Ccr5*	2.337	1.22	0.0001
ENSRNOG00000019050	*Ifit1*	2.308	1.21	0.0071
ENSRNOG00000022523	*Fkbp5*	2.300	1.20	0.00005
ENSRNOG00000001469	*Eln*	2.298	1.20	0.01635
ENSRNOG00000029301	-	2.295	1.20	0.01285
ENSRNOG00000038275	*Adam4*	2.281	1.19	0.0381
ENSRNOG00000018251	*D3ZAM0_RAT*	2.279	1.19	0.0071
ENSRNOG00000020991	*Ms4a11*	2.261	1.18	0.00935
ENSRNOG00000021433	*RGD1310376*	2.260	1.18	0.0048
ENSRNOG00000037922	*F1M6H5_RAT*	2.259	1.18	0.0019
ENSRNOG00000029805	*D3ZBF9_RAT*	2.248	1.17	0.03705
ENSRNOG00000007740	*Tacstd2*	2.246	1.17	0.01575
ENSRNOG00000033615	*NU3M_RAT*	2.235	1.16	0.0012
ENSRNOG00000020119	*Pcdha6*	2.223	1.15	0.00275
ENSRNOG00000017669	*D3ZRY1_RAT*	2.221	1.15	0.00005
ENSRNOG00000018212	*Fam108a1*	2.219	1.15	0.0001
ENSRNOG00000040195	*F1LZT0_RAT*	2.218	1.15	0.00005
ENSRNOG00000030535	*RGD1308818*	2.204	1.14	0.0031
ENSRNOG00000004874	*Flrt3*	2.196	1.13	0.00175
ENSRNOG00000010345	*RGD1559502*	2.194	1.13	0.0317
ENSRNOG00000038638	-	2.184	1.13	0.00025
ENSRNOG00000017676	*Plvap*	2.184	1.13	0.00015
ENSRNOG00000005053	*Egln3*	2.173	1.12	0.0008
ENSRNOG00000019383	*Tef*	2.162	1.11	0.0013
ENSRNOG00000032609	-	2.160	1.11	0.00215
ENSRNOG00000016219	*Vnn1*	2.156	1.11	0.00005
ENSRNOG00000019890	*Folr2*	2.153	1.11	0.0221
ENSRNOG00000015716	*Gp2*	2.141	1.10	0.00045
ENSRNOG00000001419	*Cldn15*	2.139	1.10	0.029
ENSRNOG00000028082	*Tal2*	2.139	1.10	0.04195
ENSRNOG00000037562	*Zbtb42*	2.128	1.09	0.03755
ENSRNOG00000021900	*F1M385_RAT*	2.123	1.09	0.00435
ENSRNOG00000030478	-	2.120	1.08	0.00025
ENSRNOG00000042620	*Marveld1*	2.106	1.07	0.011
ENSRNOG00000003748	*RGD1565785*	2.106	1.07	0.0244
ENSRNOG00000015024	*E9PT54_RAT*	2.097	1.07	0.00065
ENSRNOG00000015037	*Vsig1*	2.097	1.07	0.047
ENSRNOG00000031598	*Atp8b4*	2.096	1.07	0.04555
ENSRNOG00000019206	*Nupr1*	2.095	1.07	0.00085
ENSRNOG00000043866	-	2.087	1.06	0.0004
ENSRNOG00000042162	*Wfdc3*	2.086	1.06	0.00865

Table S3. *Cont.*

Gene_ID	Gene Symbol	Fold Changes	Log2	*p*-Value
ENSRNOG00000028640	*Siva1*	2.084	1.06	0.04085
ENSRNOG00000016301	*Dmrt2*	2.084	1.06	0.00965
ENSRNOG00000006986	*RGD1304624*	2.072	1.05	0.0152
ENSRNOG00000030371	*COX2_RAT*	2.069	1.05	0.03495
ENSRNOG00000018859	*P3IP1_RAT*	2.067	1.05	0.021
ENSRNOG00000043048	*LOC100188984*	2.057	1.04	0.0194
ENSRNOG00000028888	*Serpina10*	2.057	1.04	0.01745
ENSRNOG00000006767	*Cst7*	2.053	1.04	0.0475
ENSRNOG00000026644	*Glipr1*	2.052	1.04	0.00335
ENSRNOG00000022480	*Atp6v1g3*	2.052	1.04	0.00005
ENSRNOG00000024335	*Fastkd1*	2.048	1.03	0.00095
ENSRNOG00000021156	*Vegfb*	2.042	1.03	0.0008
ENSRNOG00000017912	*Atp2a3*	2.040	1.03	0.00775
ENSRNOG00000000297	*Aqp2*	2.040	1.03	0.0004
ENSRNOG00000003870	*C1qtnf2*	2.037	1.03	0.03065
ENSRNOG00000043451	*LOC100359743*	2.035	1.02	0.0001
ENSRNOG00000006663	*Usp2*	2.035	1.02	0.00155
ENSRNOG00000004660	*Fzd6*	2.034	1.02	0.00075
ENSRNOG00000009620	*Cybrd1*	2.031	1.02	0.0327
ENSRNOG00000003242	*Gulp1*	2.029	1.02	0.0002
ENSRNOG00000003927	*Cd55*	2.028	1.02	0.0013
ENSRNOG00000009848	*Il18*	2.026	1.02	0.00605
ENSRNOG00000001187	*Oasl*	2.025	1.02	0.0409
ENSRNOG00000018943	*Tnnc1*	2.021	1.02	0.00125
ENSRNOG00000033745	*LOC100360824*	2.017	1.01	0.0025
ENSRNOG00000016413	*Pstpip1*	2.011	1.01	0.04135
ENSRNOG00000019943	*Slc7a6*	2.007	1.00	0.0144
ENSRNOG00000019767	*Amigo1*	2.003	1.00	0.0195
Downregulated: 185 genes				
ENSRNOG00000008015	*Fos*	0.499	−1.00	0.0039
ENSRNOG00000001645	*RGD1565927*	0.495	−1.02	0.005
ENSRNOG00000018752	*Clcf1*	0.491	−1.03	0.04025
ENSRNOG00000025216	*Alkbh6*	0.487	−1.04	0.01425
ENSRNOG00000018484	*Plk3*	0.487	−1.04	0.01585
ENSRNOG00000019422	*Egr1*	0.484	−1.05	0.00015
ENSRNOG00000010415	*Atxn7l1*	0.484	−1.05	0.01575
ENSRNOG00000009734	*Akr1b8*	0.483	−1.05	0.0003
ENSRNOG00000029726	*Gstm1*	0.483	−1.05	0.00055
ENSRNOG00000010906	*Ccl5*	0.483	−1.05	0.02785
ENSRNOG00000001623	*Znf295*	0.482	−1.05	0.00145
ENSRNOG00000012420	*Bcl9l*	0.481	−1.06	0.0013
ENSRNOG00000001414	*Serpine1*	0.481	−1.06	0.0005
ENSRNOG00000018321	*Sytl3*	0.481	−1.06	0.00455
ENSRNOG00000033571	*F1M1A3_RAT*	0.481	−1.06	0.04275

Table S3. *Cont.*

Gene_ID	Gene Symbol	Fold Changes	Log2	*p*-Value
ENSRNOG00000009225	*Copz2*	0.480	−1.06	0.0465
ENSRNOG00000043439	*RGD1562665*	0.476	−1.07	0.0493
ENSRNOG00000004100	*Trib1*	0.476	−1.07	0.0006
ENSRNOG00000014061	*Dusp5*	0.475	−1.07	0.00115
ENSRNOG00000016250	*Ammecr1l*	0.474	−1.08	0.00005
ENSRNOG00000024433	*Fbxl7*	0.473	−1.08	0.04815
ENSRNOG00000032508	*Acot5*	0.473	−1.08	0.0174
ENSRNOG00000009253	*Igsf9b*	0.472	−1.08	0.0232
ENSRNOG00000019763	*Mlph*	0.472	−1.08	0.0003
ENSRNOG00000007657	*Col27a1*	0.470	−1.09	0.00645
ENSRNOG00000001378	*IQCD_RAT*	0.469	−1.09	0.00135
ENSRNOG00000014414	*Qrfpr*	0.469	−1.09	0.048
ENSRNOG00000001578	*Hoxd4*	0.467	−1.10	0.0186
ENSRNOG00000015873	*Spns3*	0.466	−1.10	0.0069
ENSRNOG00000033433	*Csrnp1*	0.465	−1.10	0.00155
ENSRNOG00000030492	*LOC100364577*	0.463	−1.11	0.0003
ENSRNOG00000011026	*Pqcp*	0.463	−1.11	0.00095
ENSRNOG00000016737	*Tcerg1l*	0.461	−1.12	0.01675
ENSRNOG00000005359	*Csrnp3*	0.458	−1.13	0.0078
ENSRNOG00000005255	*F1LY07_RAT*	0.455	−1.14	0.03315
ENSRNOG00000002983	*Nfix*	0.454	−1.14	0.01895
ENSRNOG00000017560	*Mdk*	0.454	−1.14	0.00575
ENSRNOG00000010280	*Pde8b*	0.452	−1.15	0.02625
ENSRNOG00000008553	*Mthfr*	0.450	−1.15	0.00025
ENSRNOG00000019140	*Banp*	0.449	−1.16	0.00325
ENSRNOG00000029172	*LOC100361944*	0.447	−1.16	0.01465
ENSRNOG00000016975	*Pxmp4*	0.445	−1.17	0.0002
ENSRNOG00000030897	*F1LVB2_RAT*	0.443	−1.17	0.03895
ENSRNOG00000014117	*Hmox1*	0.438	−1.19	0.00495
ENSRNOG00000013835	*Timm8a2*	0.437	−1.20	0.03585
ENSRNOG00000013092	*D3ZER0_RAT*	0.435	−1.20	0.0012
ENSRNOG00000013408	*Npas2*	0.433	−1.21	0.00085
ENSRNOG00000010812	*Osbpl6*	0.433	−1.21	0.0014
ENSRNOG00000003494	*Ppfia4*	0.431	−1.21	0.00015
ENSRNOG00000007607	*Nr4a1*	0.430	−1.22	0.0003
ENSRNOG00000019689	*Vwf*	0.428	−1.22	0.0001
ENSRNOG00000009519	*Ttc39a*	0.424	−1.24	0.03655
ENSRNOG00000003348	*Rasd1*	0.424	−1.24	0.01495
ENSRNOG00000021403	*Rhob*	0.423	−1.24	0.00015
ENSRNOG00000007793	*Pnrc1*	0.423	−1.24	0.00005
ENSRNOG00000037149	*RGD1562344*	0.422	−1.24	0.0437
ENSRNOG00000014424	*F1M4P5_RAT*	0.422	−1.25	0.00055
ENSRNOG00000007822	*Vgll4*	0.418	−1.26	0.00015

Table S3. *Cont.*

Gene_ID	Gene Symbol	Fold Changes	Log2	*p*-Value
ENSRNOG00000021086	*Dtx4*	0.416	−1.27	0.00435
ENSRNOG00000037307	*Spata22*	0.416	−1.27	0.00005
ENSRNOG00000019673	*Zfp36*	0.411	−1.28	0.00005
ENSRNOG00000033893	*Cacna1h*	0.410	−1.29	0.0037
ENSRNOG00000011483	*S100a9*	0.403	−1.31	0.0104
ENSRNOG00000034195	*Bpi*	0.402	−1.31	0.04485
ENSRNOG00000021752	*Foxq1*	0.402	−1.32	0.0047
ENSRNOG00000033100	-	0.401	−1.32	0.0287
ENSRNOG00000015801	*Spns2*	0.400	−1.32	0.00005
ENSRNOG00000011585	*Fat3*	0.398	−1.33	0.0293
ENSRNOG00000025691	*Pla2g7*	0.396	−1.33	0.00005
ENSRNOG00000008187	*Ubash3b*	0.396	−1.34	0.00035
ENSRNOG00000031090	*RT1-CE7*	0.396	−1.34	0.002
ENSRNOG00000015124	*Gpam*	0.394	−1.34	0.00005
ENSRNOG00000021745	*Bhlhe22*	0.393	−1.35	0.00535
ENSRNOG00000021110	*Mllt11*	0.387	−1.37	0.0071
ENSRNOG00000031686	*Hba2*	0.380	−1.40	0.00035
ENSRNOG00000008976	*F1M014_RAT*	0.379	−1.40	0.0313
ENSRNOG00000007412	*Dok1*	0.377	−1.41	0.02345
ENSRNOG00000039874	*D4A8E2_RAT*	0.375	−1.42	0.01405
ENSRNOG00000015036	*Ctgf*	0.374	−1.42	0.00005
ENSRNOG00000004585	*Tmtc2*	0.374	−1.42	0.0326
ENSRNOG00000014350	*Cyr61*	0.366	−1.45	0.00005
ENSRNOG00000037667	*RGD1561147*	0.365	−1.45	0.00895
ENSRNOG00000014338	*Slc25a25*	0.363	−1.46	0.00005
ENSRNOG00000009803	*LOC100362176*	0.359	−1.48	0.0267
ENSRNOG00000018275	*Errfi1*	0.359	−1.48	0.00005
ENSRNOG00000014576	*F1M4N6_RAT*	0.356	−1.49	0.00005
ENSRNOG00000000503	*Ppard*	0.347	−1.53	0.00005
ENSRNOG00000023532	*Ankfn1*	0.345	−1.54	0.0069
ENSRNOG00000027770	*F1LN45_RAT*	0.343	−1.54	0.0296
ENSRNOG00000000609	*Ipmk*	0.343	−1.54	0.00005
ENSRNOG00000015740	*Rnf125*	0.340	−1.56	0.0001
ENSRNOG00000015148	*Erc2*	0.336	−1.58	0.00185
ENSRNOG00000003300	*Btg2*	0.329	−1.60	0.00005
ENSRNOG00000023465	*LOC500300*	0.328	−1.61	0.00005
ENSRNOG00000010047	*LOC100363484*	0.328	−1.61	0.00255
ENSRNOG00000007830	*Apold1*	0.326	−1.62	0.0001
ENSRNOG00000016885	*Klf6*	0.324	−1.63	0.00005
ENSRNOG00000001607	*Adamts1*	0.322	−1.63	0.00005
ENSRNOG00000014385	*Wnt2b*	0.319	−1.65	0.0092
ENSRNOG00000037206	*Ccdc77*	0.316	−1.66	0.0001
ENSRNOG00000031230	*LOC689064*	0.314	−1.67	0.0027

Table S3. *Cont.*

Gene_ID	Gene Symbol	Fold Changes	Log2	*p*-Value
ENSRNOG00000014486	*Rfx3*	0.314	−1.67	0.0007
ENSRNOG00000006627	*D3ZCZ3_RAT*	0.302	−1.73	0.00005
ENSRNOG00000019260	*D4ADQ8_RAT*	0.301	−1.73	0.0042
ENSRNOG00000005447	*RGD1311564*	0.300	−1.73	0.00005
ENSRNOG00000024506	*LOC500475*	0.300	−1.74	0.03115
ENSRNOG00000022719	*Abcb1b*	0.300	−1.74	0.00065
ENSRNOG00000029886	*Hba-a2*	0.297	−1.75	0.00005
ENSRNOG00000010332	*Nipsnap3b*	0.295	−1.76	0.00025
ENSRNOG00000029622	*Olr1668*	0.293	−1.77	0.00005
ENSRNOG00000040052	*RT1-M6-2*	0.284	−1.82	0.0072
ENSRNOG00000004500	*Myc*	0.280	−1.83	0.0002
ENSRNOG00000028548	*Ccl9*	0.272	−1.88	0.00475
ENSRNOG00000007489	*Zfp41*	0.268	−1.90	0.0193
ENSRNOG00000012747	*Spock1*	0.268	−1.90	0.0034
ENSRNOG00000011250	*Inmt*	0.266	−1.91	0.00005
ENSRNOG00000018059	*Ihh*	0.260	−1.94	0.00005
ENSRNOG00000042316	*F1LPR6_RAT*	0.256	−1.97	0.0069
ENSRNOG00000013062	*Cyp24a1*	0.248	−2.01	0.00005
ENSRNOG00000026770	*Tspyl3*	0.240	−2.06	0.0385
ENSRNOG00000033526	*Hspa1a*	0.240	−2.06	0.00005
ENSRNOG00000026293	*Jun*	0.230	−2.12	0.00005
ENSRNOG00000003977	*Dusp1*	0.227	−2.14	0.00005
ENSRNOG00000025510	*Hbb-b1*	0.225	−2.15	0.00285
ENSRNOG00000042965	*RGD1562378*	0.217	−2.20	0.0315
ENSRNOG00000034915	*5_8S_rRNA*	0.198	−2.34	0.00055
ENSRNOG00000019661	*Gdf15*	0.197	−2.35	0.00005
ENSRNOG00000033465	*Hbb*	0.196	−2.35	0.00005
ENSRNOG00000011815	*Sgk1*	0.192	−2.38	0.00005
ENSRNOG00000023896	*Dusp6*	0.181	−2.46	0.00005
ENSRNOG00000002607	*LOC100361122*	0.176	−2.50	0.0004
ENSRNOG00000023509	*Irs2*	0.172	−2.54	0.00005
ENSRNOG00000013090	*Gadd45g*	0.166	−2.59	0.00005
ENSRNOG00000014448	*Arntl*	0.166	−2.59	0.00005
ENSRNOG00000023316	*Grhl1*	0.166	−2.59	0.01655
ENSRNOG00000003745	*Atf3*	0.165	−2.60	0.00005
ENSRNOG00000043362	*F1M6M4_RAT*	0.160	−2.64	0.0018
ENSRNOG00000028907	*Ugt2b7*	0.148	−2.76	0.00005
ENSRNOG00000020057	*Tex101*	0.096	−3.38	0.00155
ENSRNOG00000010094	*Kcnmb2*	0.081	−3.63	0.04425
ENSRNOG00000011668	*Nfil3*	0.068	−3.87	0.00005
ENSRNOG00000029543	*Cish*	0.056	−4.17	0.00005
ENSRNOG00000029113	*Olr1331*	0.029	−5.09	0.0443
ENSRNOG00000012067	*Fam111a*	0.023	−5.47	0.00005

Table S3. *Cont.*

Gene_ID	Gene Symbol	Fold Changes	Log2	*p*-Value
ENSRNOG00000003537	*Spta1*	0.021	−5.54	0.00005
ENSRNOG00000037923	*Dmrtc1c*	8.35×10^{-3}	−6.90	0.00005
ENSRNOG00000033713	*F1LWY7_RAT*	1.26×10^{-4}	−12.96	0.00005
ENSRNOG00000022483	*Trim50*	1.23×10^{-4}	−12.99	0.00005
ENSRNOG00000009240	*LOC690096*	1.08×10^{-4}	−13.18	0.00805
ENSRNOG00000043482	*D4A5K2_RAT*	9.82×10^{-5}	−13.31	0.00805
ENSRNOG00000033321	*RGD1559962*	9.33×10^{-5}	−13.39	0.00005
ENSRNOG00000006857	*RGD1311080*	8.99×10^{-5}	−13.44	0.00005
ENSRNOG00000009875	*Akr1b7*	8.43×10^{-5}	−13.53	0.00005
ENSRNOG00000017629	-	8.16×10^{-5}	−13.58	0.00805
ENSRNOG00000013038	-	7.68×10^{-5}	−13.67	0.00005
ENSRNOG00000034522	*SNORA73*	7.08×10^{-5}	−13.79	0.03915
ENSRNOG00000027689	-	6.25×10^{-5}	−13.97	0.0182
ENSRNOG00000018787	*LOC100364844*	6.20×10^{-5}	−13.98	0.0218
ENSRNOG00000033916	*LOC100363012*	5.98×10^{-5}	−14.03	0.0218
ENSRNOG00000045340	*RNaseP_nuc*	5.10×10^{-5}	−14.26	0.0218
ENSRNOG00000038310	*D3ZEP5_RAT*	4.95×10^{-5}	−14.30	0.00115
ENSRNOG00000043005	*F1LTX7_RAT*	3.92×10^{-5}	−14.64	0.00115
ENSRNOG00000004329	-	3.57×10^{-5}	−14.77	0.00115
ENSRNOG00000029121	*LOC100363638*	3.27×10^{-5}	−14.90	0.00005
ENSRNOG00000033179	*Olr1326*	2.54×10^{-5}	−15.26	0.00005
ENSRNOG00000035270	*SNORA41*	1.47×10^{-5}	−16.05	0.0258
ENSRNOG00000035302	*SNORA42*	1.26×10^{-5}	−16.27	0.04705
ENSRNOG00000041965	-	3.24×10^{-6}	−18.24	0.0373
ENSRNOG00000043646	-	3.22×10^{-6}	−18.24	0.0218
ENSRNOG00000034932	*SNORD87*	3.11×10^{-6}	−18.30	0.0373
ENSRNOG00000035637	*rno-mir-29b-2*	3.04×10^{-6}	−18.33	0.0373
ENSRNOG00000041989	-	2.94×10^{-6}	−18.37	0.0373
ENSRNOG00000040819	*SNORD24*	2.72×10^{-6}	−18.49	0.0373
ENSRNOG00000041744	-	2.55×10^{-6}	−18.58	0.0373
ENSRNOG00000035498	*rno-mir-206*	2.40×10^{-6}	−18.67	0.0373
ENSRNOG00000041354	-	2.29×10^{-6}	−18.74	0.0345
ENSRNOG00000040389	-	2.28×10^{-6}	−18.74	0.0373
ENSRNOG00000041197	-	2.25×10^{-6}	−18.76	0.0373
ENSRNOG00000040911	*SNORD73*	1.89×10^{-6}	−19.01	0.00705
ENSRNOG00000040663	*SNORD65*	1.74×10^{-6}	−19.13	0.0373
ENSRNOG00000035263	*SNORD25*	1.54×10^{-6}	−19.31	0.0373
ENSRNOG00000036475	-	1.50×10^{-6}	−19.35	0.00805
ENSRNOG00000043737	-	1.14×10^{-6}	−19.74	0.0373
ENSRNOG00000041583	-	8.07×10^{-7}	−20.24	0.0373
ENSRNOG00000041459	-	6.59×10^{-7}	−20.53	0.0218

Genes that changed by RPKM > 0.3 and ≧2-fold differences between L-NAME-treated offspring *vs.* control at 3 months of age. Genes are sorted by fold changes in descending order.
